# MSCNet: Efficient and accurate semantic segmentation of LiDAR data using Multi-scale Convolution

**DOI:** 10.1371/journal.pone.0345761

**Published:** 2026-04-07

**Authors:** Xuewen Feng, Aiming Wang, Guoying Meng, Yiyang Xu, Jie Yang, Xiaohan Cheng, Yu Feng

**Affiliations:** 1 School of Mechanical and Electrical Engineering, China University of Mining and Technology -Beijing, Beijing, China; 2 Beijing China Coal Mine Engineering Co., Ltd, Beijing, China; 3 Chinese Institute of Coal Science, Beijing, China; Shandong Agricultural University, CHINA

## Abstract

In autonomous driving and intelligent robotics, the semantic information of LiDAR (Light Detection and Ranging) sensor data is crucial for understanding the surrounding environment. However, directly operating on point clouds is computationally expensive. To address this, some researchers have projected three-dimensional LiDAR data onto a two-dimensional spherical range view and used two-dimensional convolutional neural networks to segment the projected images. While the results are promising, many of these models are structurally complex, with high spatiotemporal complexity, which makes them unsuitable for real-time applications. To solve these issues, this paper proposes a multi-scale LiDAR data semantic segmentation method, MSCNet, with fewer parameters and higher segmentation accuracy. In the encoding phase, a single-channel multi-scale feature fusion block is introduced to alleviate the distribution differences between input channels. To obtain more stable local features, multi-scale dilated convolution residual blocks are designed to encode information from different receptive fields. To quickly capture global features, a pyramid pooling module is introduced. Experimental results on the SemanticKITTI, SemanticPOSS, and Pandaset datasets show that MSCNet achieves a good balance between parameter, accuracy, and running time. Particularly on the SemanticPOSS and Pandaset datasets, MSCNet achieves the best performance. Under the same parameter conditions, this method outperforms existing point cloud-based and projection-based methods.

## Introduction

The ability to understand the environment is one of the primary tasks for developing self-driving vehicles and robots. To realize three-dimensional(3D) environment perception, light detection and ranging(LiDAR) sensors play a crucial role in autonomous vehicles since they measure distance accurately [1–5]. However, large point clouds generated by LiDAR sensors are difficult to understand due to their disorder and irregularity.

In recent years, scholars have devised several feature extraction modules for unstructured point clouds and used the modules to construct network models [6–9]. These point-based methods perform well in component segmentation and small indoor scenes. However, [10] showed that such methods performed poorly in terms of efficiency and accuracy when applied to outdoor scenes. Therefore, some scholars have attempted to project 3D LiDAR data onto the 2D plane and then use segmentation models of conventional images [11–13] to obtain the semantic information in the 2D plane. However, LiDAR data is different from image data, resulting in poor accuracy using the original image segmentation model. To improve the accuracy, [14–17] redesigned the feature extraction module and network structure for the LiDAR data.

Projection-based 2D representations, which map raw point clouds onto a structured plane, offer a practical and widely used solution for LiDAR semantic segmentation. Unlike RGB images with three correlated colour channels (R/G/B), LiDAR data comprise heterogeneous attributes (e.g., 3D coordinates, intensity, and range) that describe complementary aspects of the same object and exhibit distinct channel-wise distributions. To address this issue, we propose two modules and develop a lightweight MSCNet. As shown in Fig 2, the Single Channel Multi-Scale Feature (SCMF) Extraction module extracts multi-scale features from each channel independently to alleviate inter-channel distribution mismatch. The backbone employs a Dilated Convolutional Residual (DCR) module to expand the receptive field and capture multi-scale context efficiently. In addition, a pyramid pooling module is integrated to aggregate global context. Finally, a post-processing step assigns semantic labels to occluded points.

**Fig 1:**
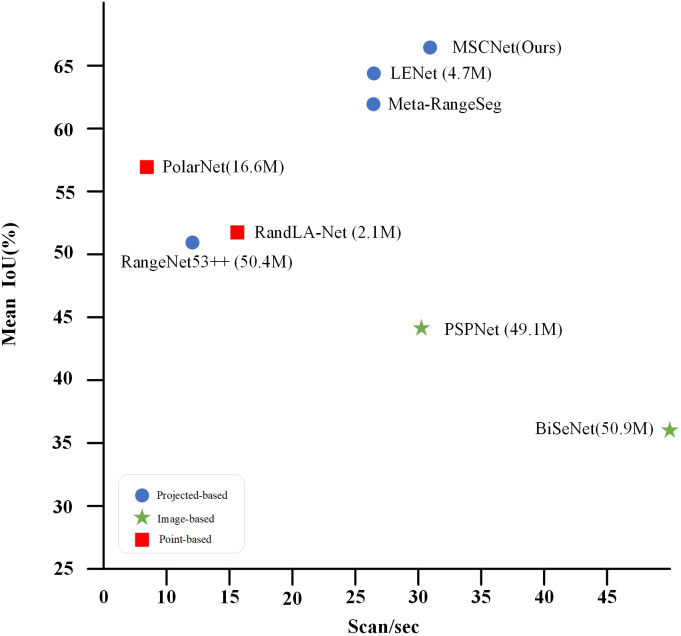
Accuracy, number of parameters, and speed of 3D LiDAR semantic segmentation in the SemanticKITTI test set [10]. Blue circles indicate projection-based methods, green pentagons indicate image-based methods, and red squares indicate point-based methods. The total number of network parameters in millions is shown in parentheses. In comparison with previous methods, the MSCNet method proposed in this paper achieves the best trade-off between accuracy, number of parameters, and speed.

In this work, we use a simple projection method to map LiDAR data to regular range image data and use this data as input to the proposed MSCNet model, which is an end-to-end fully convolutional network(FCN). The MSCNet model can be trained and tested on a single RTX 3090 with 24G of RAM, and the model achieves the best balance between accuracy, parameters, and speed (see Fig 1). The main contributions of this paper are listed below:

1. We propose a single-channel multi-scale feature fusion block (SCMFBlock) to better capture diverse features of the same object. SCMFBlock extracts multi-scale features from each input channel and fuses them across channels, enhancing feature representation.
2. To extract point features, neighborhood features, and symmetry features more efficiently, we propose a novel dilated convolutional residuals block (DCRBlock) as the backbone of the network, which uses dilation convolution to expand the perceptual field and reduce the module parameters.
3. We designed a novel FCN model named MSCNet, which uses SCMFBlock at the first layer, DCRBlock as the backbone, and, to obtain global features quickly, a pyramid pooling module. In the public datasets SemanticKITTI, SemanticPoss and PandaSet tests, MSCNet has a low number of parameters and high accuracy compared to network structures of similar size.

## Related work

With the popularity of autonomous driving, there has been an increasing amount of research on semantic scene understanding. Fast acquiring LiDAR semantic information not only improves the accuracy of mapping and localisation, but also has important implications for local environment perception [18,19]. This section briefly describes existing approaches to the semantic understanding of LiDAR point clouds. For the unstructured nature of point cloud data, a unique feature extractor must be designed and used to construct a model. The pioneering models are PointNet [6] and PointNet++ [7], where PointNet uses a multilayer perceptron (MLP) to extract features and uses a maximum pooling operation to achieve invariant point cloud feature alignment. The method does not consider the local features of the point cloud. However, PointNet++ adds a local feature capture module to complete the PointNet model. TangentConv [20] projects local points onto the tangent plane and applies 2D convolution to them, in addition to processing 3D information directly. The above methods are mainly aimed at small-scale scenes with a limited number of points, primarily indoor scenes. In contrast, RandLA-Net [21] uses random point sampling and designs better local feature aggregation modules to preserve geometric details and achieve better performance. To alleviate the instability issues of random sampling, the PCB-RandNet [22] model is proposed. This model introduces a Polar Cylinder Balanced Random Sampling method, which ensures a more balanced distribution of the downsampled point cloud across different spatial distributions, thereby improving segmentation performance. For comparison, the PolarNet [23] coding scheme uses a polar coordinate bird’s-eye-view. It balances the points in the grid cells in the polar coordinate system, indirectly aligning the attention of the segmented network with the long-tailed distribution of points along the radial axis. However, the feature extraction modules of these methods use a large number of 1×1 convolution operations. Although the number of network parameters is low, the models are time-consuming to run and not ideal in terms of accuracy. Alternatively, point clouds can be structured into voxels, and voxel features can be extracted using 3D convolution to complete the task of point cloud semantic understanding. Although such methods are more accurate, they are also accompanied by extremely high spatio-temporal complexity, high enough to meet real-time requirements. For example, on the Tesla V100, the inference speed of SPVCNN [24] and Cylinder3D [25] is only 8.1 and 7.6 fps, respectively.

Several methods exist for mapping 3D-LiDAR data to the 2D plane, and RGB-based image segmentation methods can be applied directly to this. FCN [26] treats the segmentation task as an intensive prediction, using a full convolutional structure to complete the classification of each pixel. [27,28] use an encoder-decoder structure to obtain the class of each pixel. Although the above methods achieve good performance on RGB image segmentation, they have a small perceptual field. Therefore, the DeepLab series [12,29,30] introduced dilation convolution to obtain a larger receptive field and extract image features over several scales. The dilation convolution can enhance the receptive field without losing information. PSPNet [11] proposed a pyramid pooling module to quickly capture the global information of an image. Due to the importance of semantic segmentation for autonomous driving, several research efforts have focused on this area, such as PIDNet [31], which uses convolutions with different dilation rates and parallels or concatenates them to obtain multi-scale information gain. As a comparison, BiSeNet [13] first proposed a bi-directional segmentation network where one module processes spatial information and the other captures global information, and finally uses a novel feature fusion module to complete the segmentation task. However, due to the multimodal nature of LiDAR data, the segmentation of the projected 3D-LiDAR image using the above network was not effective.

To satisfy the requirements of autonomous driving for model speed and accuracy, scholars have proposed projection-based methods that process LiDAR data projections better than segmentation models that use RGB images directly. squeezeSeg [14] and SqueezeSegv2 [15] use SqueezeNet [32] as the backbone and post-processing using conditional random fields (CRF). These two models run fast but with low accuracy. Therefore, SqueezeSegv3 [33] proposed a spatially adaptive convolution module to address the problem of significant variation in the distribution of features in projection images at different locations, but this model is time-consuming to run. Unlike SqueezeSeg, RangeNet++ [16] used Darknet [34] as the backbone network and proposed a k-nearest-neighbor (k-NN) search for post-processing. The above projection-based models are either low in accuracy or have a significant number of parameters. Therefore, MINet [35] uses multiple paths at different scales to balance computational resources, and the network achieves a good balance in terms of the number of parameters, running speed, and accuracy. In contrast, 3D-MiniNet [36] combines 3D and 2D learning layers, learning 2D representations from the original points through a new projection that captures local and global information about the 3D data. Then, the projection is used as input to a 2D Full Convolutional Neural Network (FCNN) and results in 2D semantic segmentation. Although the projection-based approach is practical, the multiscale nature of multimodal information is not considered in the above approach. Therefore, in this work, we designed extractors and fusion modules for multimodal features to achieve a balance of parameters, speed, and accuracy.

## Methods

The goal of the approach proposed in this paper is to balance the number of parameters, running time, and accuracy of the semantic segmentation model to guarantee real-time and accurate perception of the environment. In this section, our approach is described in detail. First, we briefly introduce the point cloud projection, and then we will discuss the network architecture, loss function, and training details.

### LiDAR data projection

To ensure the real-time nature of the model, we use a spherical projection method to project the LiDAR point cloud into a spherical coordinate system to form a 2D range image. Then, the features are extracted using 2D convolution operations in computer vision to convert the point cloud segmentation task into an image segmentation problem. Thus, the point cloud is first projected onto a 2D plane with the following equation:


(uv)=(12[1−arctan(y,x)π−1]W[1−(arcsin(zd−1)+fup)f−1]H) (1)

where (u,v) are angular coordinates, (x,y,z) are raw LiDAR point, H and W are the height and width of the desired projected 2D map, *d* represents the depth of each point as $d=\sqrt{x^{2}+y^{2}+z^{2}}$, *f* represents the vertical field of view of the sensor as $f=f_{up}+f_{down}$.

Using equation (1), the input data of [W×H×C] is obtained, where W and H can be of arbitrary size, in this paper we take *W* = 2048*px*, *H* = 64*px* and the number of channels *C*=(*x*,*y*,*z*,*d*,*r*), *d* represents the depth, *r* is the intensity read by the sensor. In the next subsections, a new neural network structure and its module will be explained, and the model’s and the module’s validity will be verified using experimental results and ablation experiments, respectively.

### MSCNet model architecture

**Fig 2:**
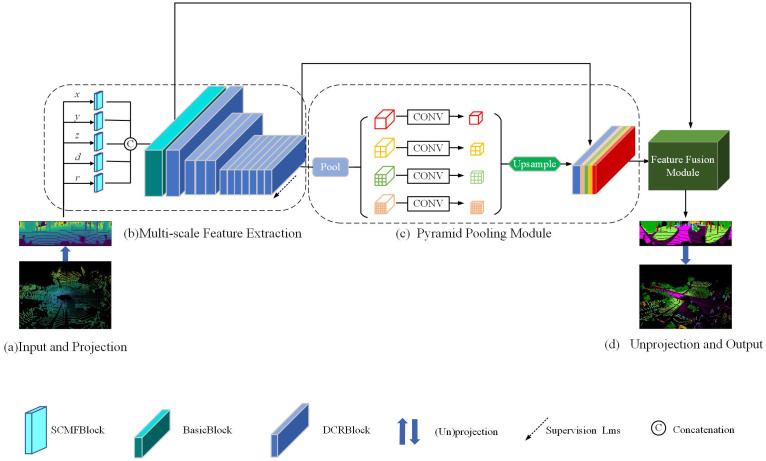
The architecture of our proposed MSCNet model. Given LiDAR Data, we first use spherical projection to get a range image(a), and SCMF block, BasicBlock, and RDC block are applied to build multi-scale feature acquisition models(b), where the dashed arrows indicate the type of supervision. Then, the pyramid parsing module is used to obtain different sub-regional representations, which are upsampled, and DCRBlock output features are concatenated to form the final feature representation, which contains both local and global information(c). Finally, the representation is fed into the Feature Fusion Module to obtain a pixel-by-pixel prediction, and a point-by-point prediction is obtained using the inverse projection(d). The different blocks are illustrated in Fig 3, and Feature Fusion Module is illustrated in Fig 4.

**Fig 3:**
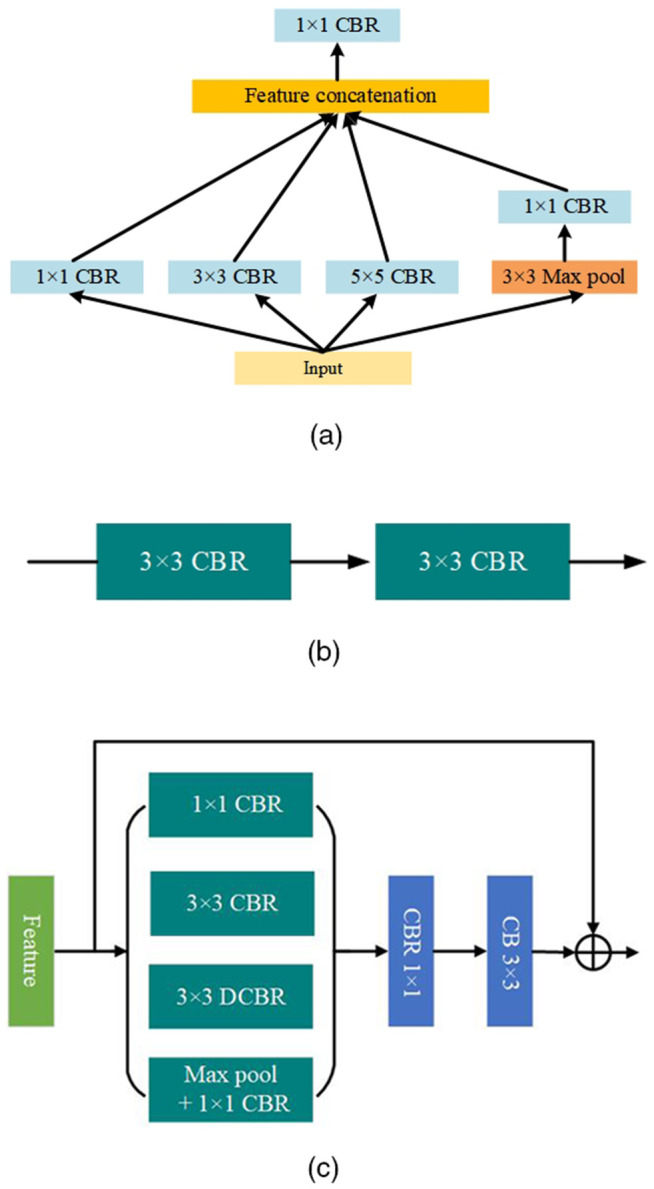
Illustrations of SCMFBlock (a), BasicBlock (b) and DCRBlock (c). where CBR = Conv + BN + LeakyReLU, DCBR is a CBR with a dilation rate of 2. 3×3 and 1×1 denote the convolution kernel size.

The structure of MSCNet is shown in Fig 2. The input to the model is a 2D projection of the LiDAR point cloud, as described in Methods section. The first layer consists of five parallel SCMFBlock modules (see Fig 3(a)), which complete the information calibration for each channel and use the BasicBlock module (see Fig 3(b)) to extract the corrected multi-modal information. The backbone network is constructed using the DCRBlock module (see Fig 3(c)) to extract point features and multi-scale neighborhood feature extraction. To obtain global information quickly, different scale pyramidal pooling modules are utilized to improve the network operation and convergence speed. The final 2D prediction at the original resolution is generated in the feature fusion module and back-projected into 3D space. Next, each module is described in detail.

**Single Channel Mutil-scale Feature Fusion Block (SCMFBlock).** Unlike the RGB image representation, the range image contains (x,y,z,d,r) channels of five different modalities. Most projection-based segmentation methods are homogeneous across modalities at scale. However, we show that it is more efficient to use SCMFBlock (see Fig 3(a)) to extract multi-scale features for each channel individually. Specifically, multi-scale features are first extracted for a single channel using three different scales of CBR blocks and Maxpool, where CBR represents a sequential combination of convolution, normalization, and activation function operations. Each channel is then mapped to a separate feature space using 1×1 convolution of the multi-scale information. This step can be considered a feature calibration for each channel before fusion. It is worth noting that each channel passes through a separate SCMFBlock. After passing through five SCMFBlocks in parallel, the features of each channel are concatenated and sent to the BasicBlock (shown in Fig 3(b), consisting of two layers of 3×3 CBR superimposed) to extract features. The number of channels output from the SCMFBlock is all 4, so the computation is small.

**Dilated Convolutional Residual Block (DCRBlock).** The receptive field plays a crucial role in the extraction of spatial features. To obtain more descriptive spatial features, a straightforward approach is to increase the size of the convolution kernel. However, this has the disadvantage that the number of parameters will increase dramatically as the depth of the model increases. Therefore, this paper proposes a DCRBlock to obtain a larger perceptual field. As shown in Fig 3(c), this module extracts point features using 1×1 CBR, multi-scale neighborhood features using 3×3 CBR and 3×3 DCBR, and symmetry features using a Maxpool, where DCBR denotes the sequential operation of dilation convolution, normalization, and activation function with a dilation rate of 2. This operation reduces the convolution parameters while expanding the perceptual field. Further, we concatenate each block to extract features and apply 1×1 CBR to fuse the various features so that the network can obtain different information about the receptive field. Finally, feature extraction or downsampling is completed using 3×3 convolution, and the features are fused using residual.

**Pyramid Pooling Module(PPM).** After the multi-scale feature extraction is complete, the PPM is used to obtain global features quickly. The PPM [11] incorporates four pyramid-scale features, as shown in Fig 2(c). The coarsest level highlighted in red is global pooling to generate a single bin output. The next pyramid level divides the feature map into different sub-regions, forming a pooled representation of the different locations. The output at different levels of the pyramid pooling module contains feature maps of different sizes. To maintain the weight of the global features, we use a 1×1 convolutional layer after each pyramid level. Suppose the size of the pyramid level is *N*. In that case, the dimensionality of the contextual representation is reduced to $\frac{1}{N}$ of the original dimensionality. Then the lower-dimensional feature maps are upsampled directly to obtain features of the same size as the original feature maps by bilinear interpolation. Finally, the features at different levels are concatenated as the final pyramid set of global features.

**Fig 4:**
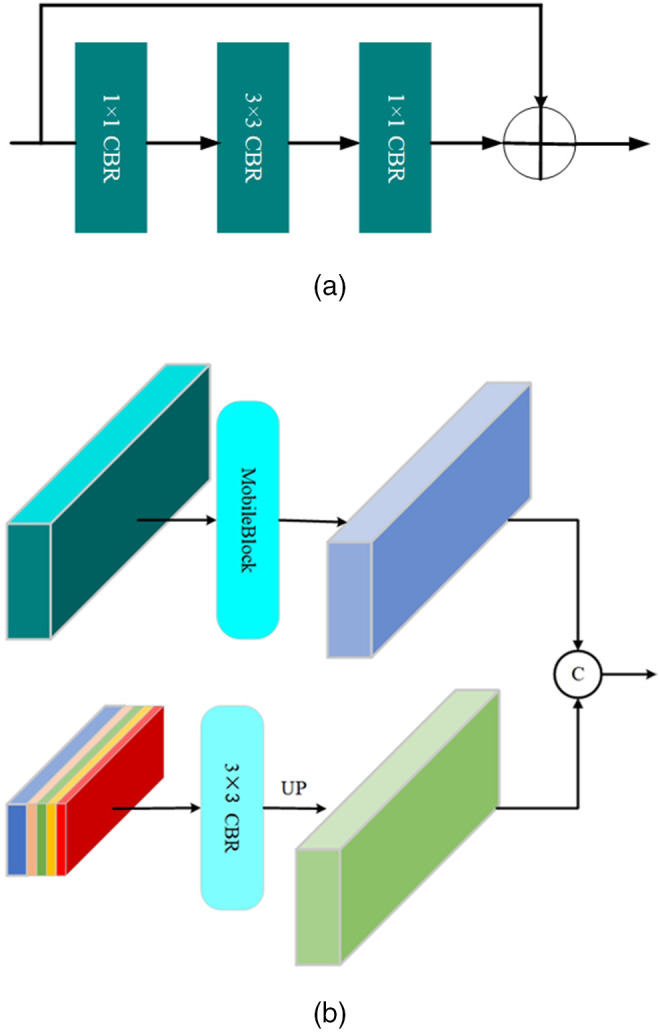
Architecture of the Decoder Module: (a) MobileBlock; (b) Feature Fusion Module.

**Feature Fusion Module(FFM).** Fig 4(b) illustrates how the Feature Fusion Module (FFM) integrates multi-scale features to assign semantic labels to each pixel in range images. The top half of Fig 4(b) receives input from the BasicBlock module shown in Fig 2, where the MobileBlock (depicted in Fig 4(a)) extracts detailed spatial features. Specifically, the MobileBlock first applies a 1 × 1 Convolution followed by Batch normalization and ReLU (CBR) to reduce channel dimensionality. It then employs a 3 × 3 CBR to capture local neighborhood information. Finally, a second 1 × 1 CBR further refines the extracted fine-grained features before they are passed into the FFM. The bottom half of Fig 4(b) shows the multi-scale concatenated feature extracted using PPM, which was processed through a convolution block containing 3×3 convolution, batch normalization, and LeakReLU activation, and upsampled to the original resolution. Finally, the data classification is completed by concatenating the features processed by the two modules.

### Loss Function

The imbalance of classes in the dataset poses a challenge for model training. In the case of bicycles and persons, for example, they have a small amount of data compared to cars and roads, which makes the network more biased towards the classes with more data in the training data, resulting in poorer network performance.

To address the class imbalance problem, this paper divides the loss function into two parts: the middle layer and the output layer supervision. Supervision of the middle layer features of the network is useful for model optimization in the experiment, and we utilize the standard weighted cross-entropy loss [16] as semantic supervision:


$$L_{s}\left(y_{c},{\hat{y}}_{c}\right)=-\sum_{c=1}^{C}w_{c}y_{c}log({\hat{y}}_{c})$$ (2)

where *y*_*c*_ and ${\hat{y}}_{c}$ denote the true and predicted category labels respectively, *f*_*c*_ denotes the frequency of class *c*, and $w_{c}=\frac{1}{log\left(f_{c}+\varepsilon \right)}$ denotes the weight of class *c*, i.e., the larger the *f*_*c*_ the smaller the *w*_*c*_, which can handle imbalanced data, e.g., a large number of points in the dataset represented by class road means a small *w*_*c*_, while a small number of points in class person means a relatively large *w*_*c*_. In addition, we use weighted cross-entropy loss Ls at the middle layer(*L*_*ms*_) and the output layer(*L*_*os*_), indicated by the dashed arrows in Fig 2(b) for *L*_*ms*_. As we will show in our experiments, this intermediate supervision improves training and increases accuracy. However, adding this semantic supervision to the underlying paths did not help much, as the resolution of the underlying paths was too low.

Besides the weighted cross-entropy, a Lovász-Softmax loss is added at the network’s end to maximize the mean intersection over union (mIoU). The mIoU metric is commonly used for segmentation performance evaluation. However, due to the discrete, non-derivative nature of IoU, it cannot be used as a direct loss. In [37], the mIoU calculation is improved using the Lovász function to obtain the Lovász-Softmax loss (*L*_*ls*_),which can be expressed as follows:


$$L_{ls}=\frac{1}{C}\sum_{c=1}^{C}\overset{―}{△J_{c}}(m(c))$$ (3)

$$\begin{array}{c} m_{i}(c)=\left\{ \begin{array}{cc} & 1-x_{i}(c)\;\text{ if }c=y_{i}(c)\\ & x_{i}(c)\;\;\text{otherwise} \end{array}\right. \end{array}$$ (4)

where $\overset{―}{△J_{c}}$ is defined as the Lovász extension of the Jaccard index, *C* denotes the number of categories, $x_{i}\left(c\right)\in \left[0,1\right]$ and $y_{i}\left(c\right)\in \left\{-1,1\right\}$ denote for class *c* at pixels *i* the predicted probability and ground-truth label, respectively.

Finally, the total loss function of the MSCNet is a linear combination of two end losses and an intermediate layer loss function, as follows:


$$L=L_{os}+L_{ls}+\lambda L_{ms}$$ (5)

In equation (5), *L*_*os*_ represents the supervision loss function applied to intermediate layers, *L*_*ms*_ denotes the supervision loss function applied to the output layer, and λ is a hyperparameter.

### Optimizer And Regularization

We utilized AdamW as the optimizer, setting the learning rate to 0.0001 and the weight decay coefficient to 0.005. Unlike the original Adam, AdamW decouples weight decay from the gradient update step, ensuring that regularization does not interfere with the adaptive learning rate mechanism. This strategy yields more stable training dynamics and improves the model’s generalization performance.To mitigate overfitting, we applied data augmentation before projecting point clouds into depth views. These augmentations include random rotations or translations, flips around the y-axis, and random point dropout, each applied with a 0.5 probability independently. Such augmentation techniques enhance model robustness by introducing diverse geometric variations.

For the SemanticKITTI dataset, we used a batch size of 6 and adopted a one-cycle learning rate schedule, training the model for 30 epochs. Input dimensions for the projection were set to W = 2048, H = 64 to align with existing range-view-based methods.On the SemanticPOSS dataset, we maintained the same data augmentation and training strategy but adjusted the input projection size to W = 1800,H = 40, and trained the MSCNet model for 60 epochs.The PandaSet configuration matched that of SemanticKITTI.

### Post-processing

The pixel label is assigned to multiple LiDAR points during the back-projection process, which leads to the misclassification of target edges. The knn-based post-processing method proposed by RangeNet++ [16] is used for this problem, which sets a small window around the corresponding image pixel and uses the value domain of the (*u*, *v*) neighborhood points representing the Euclidean distance in 3D space to quickly complete the back-projection results post-processing (running time within 7ms on GPU acceleration).

Note that this post-processing is only applied to the network output during inference and has no effect on learning.

## Experiments

### Experimental settings

To validate the MSCNet network performance, we chose three datasets for our experiments.

1. SemanticKITTI: The dataset provides semantic labels for each point. It consists of 21 sequences with over 43,000 scans, with sequence files 00–10 scanned over 21,000 times, and the remaining sequence 11–21 scanned files used for the test set. During training, sequence 08 was used as the validation set, and 00–07, 09, and 10 as the training set. The ablation experiments were performed using the validation set for functional module verification. For the test set, we performed online tests and compared them to state-of-the-art models with similar parameter numbers.
2. SemanticPOSS: This dataset, collected at Peking University, is stored in the same format as SemanticKITTI and contains 2988 complex LiDAR scans, with more moving objects and smaller objects (more complex than the urban environment). The dataset contains six sequences from 00 to 05, with 02 used as the test set and the others as the training set in our experiments.
3. Pandaset: The dataset was acquired on two routes in Silicon Valley, using both spinning and solid-state LiDARs to acquire point cloud data, with 16000 LiDAR scans. For the experiments in this paper, data from the rotating LiDAR was used, with 70% of the data used for training and the remainder for testing. As suggested, similar classes will be merged, and rare classes will be removed.

We use standard mean intersection over union(mIoU) [38] to evaluate all classes:


$$mIoU=\frac{1}{C}\sum_{c=1}^{C}\frac{TP_{c}}{TP_{c}+FP_{c}+FN_{c}}$$ (6)

where *TP*_*c*_, *FP*_*c*_, and *FN*_*c*_ denote the numbers of true positive, false positive, and false negative predictions for class *c*, respectively. *C* is the number of classes.

### Quantitative results

As shown in Table 1, we systematically compare point-based models (rows 1–10), voxel-based models (rows 11–15), image-based models (rows 16–19), and projection-based models (rows 20–31), evaluating each category in terms of parameter count (M), processing speed (Scan/sec), and segmentation accuracy (mIoU). Among point-based models, most methods exhibit relatively slow segmentation speed and low accuracy on the SemanticKITTI dataset. RandLA-Net and PolarNet stand out with competitive speed and accuracy, yet they come with high spatial complexity. Notably, although PolarNet achieves high accuracy, its parameter count exceeds 16M, leading to substantial computational overhead. For voxel-based methods, the inference speeds of Cylinder3D V2 and JS3C-Net are 5.9 scans/sec and 2.1 scans/sec, respectively, which fall short of real-time processing requirements. In contrast, MSCNet outperforms point-based models across parameter size, runtime efficiency, and segmentation accuracy, demonstrating superior overall performance.

**Table 1:** Evaluation Results on the SemanticKITTI Test Set(Sequences 11 to 21). Point-based Methods: Rows 1 to 10. Voxel-based Methods:Rows 11 to 15. Image-based Methods: Rows 16 to 19. Projection-based Methods: Rows 20 to 31.

Methods	car	bicycle	motorcycle	truck	other-vehicle	person	bicyclist	motorcyclist	road	parking	sidewalk	other-ground	building	fence	vegetation	trunk	terrain	pole	traffic-sign	Scan/sec	Param(M)	mIoU
Pointnet [6]	46.3	1.3	0.3	0.1	0.8	0.2	0.2	0.0	61.6	15.8	35.7	1.4	41.4	12.9	31.0	4.6	17.6	2.4	3.7	2.0	3.0	14.6
Pointnet++ [7]	53.7	1.9	0.2	0.9	0.2	0.9	1.0	0.0	72.0	18.7	41.8	5.6	62.3	16.9	46.5	13.8	30.0	6.0	8.9	0.1	6.0	20.1
SPGraph [8]	68.3	0.9	4.5	0.9	0.8	1.0	6.0	0.0	49.5	1.7	24.2	0.3	68.2	22.5	59.2	27.2	17.0	18.3	10.5	0.2	0.3	20.0
SPLATNet [39]	66.6	0.0	0.0	0.0	0.0	0.0	0.0	0.0	70.4	0.8	41.5	0.0	68.7	27.8	72.3	35.9	35.8	13.8	0.0	1.0	0.8	22.8
TangentConv [20]	86.8	1.3	12.7	11.6	10.2	17.1	20.2	0.5	82.9	15.2	61.7	9.0	82.8	44.2	75.5	42.5	55.5	30.2	22.2	0.3	0.4	35.9
RandLA-Net [21]	94.2	26.0	25.8	40.1	38.9	49.2	48.2	7.2	90.7	60.3	73.7	20.4	86.9	56.3	81.4	61.3	66.8	49.2	47.7	16	2.1	53.9
PCB-RandNet [22]	94.5	6.8	35.5	76.7	37.6	48.3	79.0	0.0	93.4	42.4	80.8	1.1	89.0	56.9	87.6	68.4	74.4	61.0	40.8	–	–	56.5
PolarNet [23]	93.8	40.3	30.1	22.9	28.5	43.2	40.2	5.6	90.8	61.7	74.4	21.7	90.0	61.3	84.0	65.5	67.8	51.8	57.5	8	16.6	54.3
GACNet [40]	94.2	16.5	32.2	47.4	39.1	48.4	27.5	9.4	90.5	61.8	74.0	24.5	89.7	60.4	83.8	47.4	68.6	51.0	58.9	–	–	54.9
TriEn-Net [41]	98.4	36.7	38.6	41.1	38.7	54.3	50.6	21.4	91.0	62.7	74.6	26.2	89.5	60.4	83.6	61.9	68.7	52.6	51.8	–	1.57	57.8
MinkNet [42]	94.3	23.1	26.2	26.1	36.7	43.1	36.4	7.9	91.1	63.8	69.7	29.3	92.7	57.1	83.7	68.4	64.7	57.3	60.1	–	–	54.3
SPVCNN-lite [24]	–	–	–	–	–	–	–	–	–	–	–	–	–	–	–	–	–	–	–	8.1		58.5
Cylinder3D V1 [25]	96.1	54.2	47.6	38.6	45.0	65.1	63.5	13.6	91.2	62.2	75.2	18.7	89.6	61.6	85.4	69.7	69.3	62.6	64.7	7.6		61.8
Cylinder3D V2 [25]	97.1	67.6	64.0	59.0	58.6	73.9	67.9	36.0	91.4	65.1	75.5	32.3	91.0	66.5	85.4	71.8	68.5	62.6	65.6	5.9		67.8
JS3C-Net [43]	95.8	59.3	52.9	54.3	46.0	69.5	65.4	39.9	88.9	61.9	72.1	31.9	92.5	70.8	84.5	69.8	67.9	60.7	68.7	2.1	–	66.0
DeepLabV3+ [12]	78.4	13.6	9.5	10.4	17.5	22.0	22.0	0.4	88.5	54.5	66.7	9.7	77.9	39.1	72.0	39.9	60.0	23.4	36.1	39	59.4	38.4
PSPNet [11]	79.6	25	26.4	17.5	24.0	34.1	28.4	7.3	90.2	58.2	70.2	19.9	79.7	43.5	74.2	43.2	61.2	23.1	37.5	31	49.1	44.4
BiSeNet [13]	76	13.4	11.9	18.3	7.6	16.4	26.0	0.5	87.6	49.9	64.2	6.5	74.7	34.7	69.7	36.8	58.0	19.6	32.3	50	50.9	37.1
DenseASPP [44]	78.1	20.5	18.2	20.0	16.6	27.8	28.9	5.7	88.5	53.3	67.5	9.3	76.3	39.6	70.0	36.8	57.7	15.9	32.4	20	23.4	40.2
SqueezeSegV2 [15]	81.8	18.5	17.9	13.4	14.0	20.1	25.1	3.9	88.6	45.8	67.6	17.7	73.7	41.1	71.8	35.8	60.2	20.2	36.3	50	1.0	39.7
RangeNet53++ [16]	91.4	25.7	34.4	25.7	23.0	38.3	38.8	4.8	91.8	65.0	75.2	27.8	87.4	58.6	80.5	55.1	64.6	47.9	55.9	12	50.4	52.2
MINet [35]	90.1	41.8	34	29.9	23.6	51.4	52.4	25.0	90.5	59.0	72.6	25.8	85.6	52.3	81.1	58.1	66.1	49.0	59.9	47	1.0	55.2
SqueezeSegV3 [33]	92.5	38.7	36.5	29.6	33.0	45.6	46.2	20.1	91.7	63.4	74.8	26.4	89.0	59.4	82	58.7	65.4	49.6	58.9	6	1.0	55.9
3D-MiniNet [36]	90.5	42.3	42.1	28.5	29.4	47.8	44.1	14.5	91.6	64.2	74.5	25.4	89.4	60.8	82.8	60.8	66.7	48.0	56.6	28	3.97	55.8
DGPolarNet [45]	92.6	18.5	38.9	51.5	21.0	55.1	66.8	9.6	93.4	58.4	79.4	20.0	90.1	55.2	86.8	51.5	75.9	62.6	39.4	–	–	56.5
CNN-LSTM [46]	92.6	45.7	49.6	48.6	30.2	53.8	74.6	9.2	90.7	23.3	75.7	17.6	90.0	51.3	87.1	60.8	75.4	63.9	41.5	11		56.9
ACPNet [47]	95.2	39.3	41.7	41.8	37.7	55.2	48.1	33.7	91.3	66.0	74.9	14.2	90.5	61.5	84.4	67.6	68.2	57.5	59.9	14		59.4
RangeViT [48]	95.4	55.8	43.5	29.8	42.1	63.9	58.2	38.1	93.1	70.2	80.0	32.5	92.0	69.0	85.3	70.6	71.2	60.8	64.7	–	–	64.0
Meta-RangeSeg [48]	93.9	50.1	43.8	43.9	43.2	63.7	53.1	18.7	90.6	64.3	74.6	29.2	91.1	64.7	82.6	65.5	65.5	56.3	64.2	26		61.0
LENet [49]	93.9	57.0	51.3	44.3	44.4	66.6	64.9	36.0	91.8	68.3	76.9	30.5	91.2	66.0	83.7	68.3	67.8	58.6	63.2	26	4.7	64.5
MSCNet(Ours)	94.7	**58.0**	50.5	46.6	**45.5**	64.8	**66.1**	**38.3**	91.9	69.1	78.1	**31.2**	**92.6**	**67.5**	85.6	67.3	65.5	60.5	63.8	30	1.6	**65.1**

In the image-based models, this paper validates the commonly used methods: PSPNet [11], DeepLabV3+ [12], BiseNet [13], and DenseASPP [44]. These models all have the number of input channels adjusted to 5, then the models are trained. Although such models are faster than the point-based methods, they have a more significant number of parameters and lower accuracy. The results show that the projected LiDAR data is different from the RGB images and that the LiDAR projected data is poorly processed directly using the segmentation framework of the images.

Then, the proposed model is compared with other projection-based methods. SqueezeSegV2 features a small number of parameters and fast inference speed but suffers from low accuracy. RangeNet++ achieves higher accuracy than SqueezeSegV2, though at the cost of significantly increased parameters and computational complexity. SqueezeSegV3, equipped with an enhanced feature extraction module, further improves accuracy but introduces considerable latency. 3D-MiniNet demonstrates a favourable trade-off between speed and accuracy; however, its improved performance relies on a larger number of parameters. In contrast, MINet [35] achieves a more balanced compromise among parameter count, accuracy, and speed. Compared to state-of-the-art models such as RangeViT[47] and Meta-RangeSeg [50], the proposed MSCNet model offers notable advantages: it has fewer parameters (1.6M) and, faster processing speed (30 scans per second). It achieves the highest IoU score (65.1%). When directly compared to LENet [49], MSCNet demonstrates superior performance in terms of parameter efficiency, inference speed, and segmentation accuracy. In terms of per-class metrics, MSCNet achieves the highest accuracy in 7 out of 19 classes. For the remaining 12 classes (e.g., car, truck, road), MSCNet’s performance is comparable to that of the best-performing method, LENet.

We also evaluated our method on the SemanticPOSS dataset and the comparison results are shown in Table 2. SemanticPOSS has a smaller dataset and sparser targets compared to SemanticKITTI, and therefore has a lower overall mIoU. However, our method achieves state-of-the-art on this dataset and improves the overall mIoU by 1.0%, with a much higher mIoU for individual targets than the comparison method.

**Table 2:** Evaluation Results on the SemanticPOSS Test Set(Sequences 02). Image-based Methods: Rows 1 to 3. Projection-based Methods: Rows 4 to 9.

Methods	person	rider	car	truck	plants	traffic sign	pole	building	fence	bike	road	mIoU
DeepLabV3 [12]	9.6	4.8	15.3	9.5	40.3	5.3	3.1	41.2	11.9	20.0	62.5	20.3
DenseASPP [11]	11.6	5.7	20.4	10.8	46.4	5.2	4.9	46.8	7.7	20.6	62.0	22.0
PSPNet [13]	9.0	4.8	17.6	10.3	44.1	5.1	3.2	45.3	5.9	20.0	63.0	20.8
SqueezeSegV2 [15]	18.4	11.2	34.9	15.8	56.3	11.0	4.5	47.0	25.5	32.4	71.3	29.8
RangeNet53++ [16]	14.2	8.2	35.4	9.2	58.1	6.8	2.8	55.5	28.8	32.2	66.3	28.9
MINet [35]	20.1	15.1	36.0	23.4	67.4	15.5	5.1	61.6	28.2	40.2	72.9	35.1
UnPNet [51]	17.7	17.2	39.2	13.8	67.0	9.5	5.8	66.9	31.1	40.5	68.4	34.3
SegUnet3D [17]	44.7	26.4	**50.7**	24.2	69.2	21.9	17.3	68.4	45.8	46.5	76.3	44.7
Meta-RangeSeg [50]	75.5	19.8	78.7	27.5	72.3	**25.6**	**32.3**	78.0	49.0	**52.9**	79.3	53.7
MSCNet(Ours)	**76.3**	**29.1**	**78.9**	**27.9**	**73.0**	25.1	31.4	**78.2**	**50.7**	50.9	**80.5**	**54.7**

To further validate the effectiveness of MSCNet, the PandaSet dataset was added for validation and its evaluation results are shown in Table 3. Our method can greatly improve small objects such as motorcycle, car, pedestriand, which are difficult to identify, and the overall performance is 3.0% better than the other methods.

**Table 3:** Evaluation Results on the Pandaset Test Set. Image-based Methods: Rows 1 to 3. Projection-based Methods: Rows 4 to 7.

Methods	vegetation	ground	Road	Lane Line	Road Mark	Sidewalk	car	truck	motorcycle	bus	bicycle	pedestrian	pylons	signs	cones	const-signs	building	mIoU
DeepLabV3 [12]	50.4	22.8	69.2	11.9	9.7	35.5	65.6	11.6	0.1	3.9	0.2	3.5	2.2	19.2	4.2	11.5	58.4	22.3
DenseASPP [11]	54.6	21.2	66.0	9.9	9.3	33.2	64.9	18.8	0.0	17.6	0.2	5.0	0.3	21.2	2.7	4.6	62.5	23.1
PSPNet [13]	45.9	18.1	65.4	8.4	6.8	28.1	60.2	15.6	0.0	6.2	0.2	2.5	0.0	19.0	0.9	4.5	55.2	19.8
SqueezeSegV2 [15]	61.0	33.0	76.4	18.6	13.5	43.5	73.5	30.8	0.0	21.8	1.7	4.9	1.1	20.5	3.3	3.7	65.4	27.8
RangeNet53++ [16]	64.1	36.3	80.8	25.1	19.8	48.8	76.0	30.7	1.0	17.2	2.0	9.8	6.3	32.7	6.7	13.0	71.0	31.8
UnPNet [51]	**78.2**	41.6	82.5	29.3	21.7	52.0	78.9	31.9	0.9	17.7	12.1	36.7	40.4	**63.7**	**19.5**	**24.9**	**83.8**	42.1
MSCNet(Ours)	73.6	**53.7**	**83.6**	**30.8**	**40.3**	**65.9**	**86.8**	**33.3**	**23.8**	**37.5**	**19.2**	**51.8**	**54.5**	20.5	18.5	3.0	83.3	**45.8**

### Qualitative results

**Fig 5:**
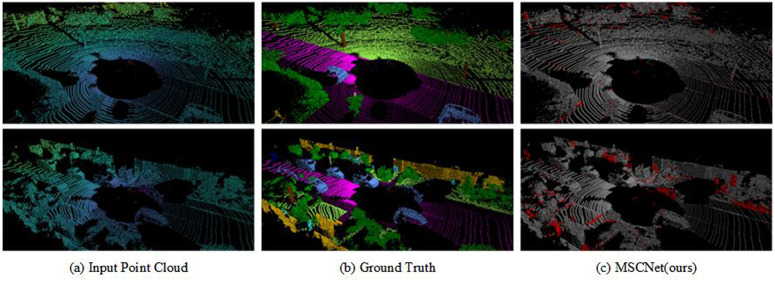
Simple qualitative results of the MSCNet model on the SemanticKITTI benchmark(valid sequence 08). Figures (a) and (b) show the raw data and corresponding segmentation labels of a LiDAR scan frame, respectively, and (c) shows the segmentation error map of our method for that scan frame (Red indicates incorrect predictions). The various colors indicate the different semantic classes: cars in blue, roads in purple, vegetation in green, and buildings in yellow.

**Fig 6:**
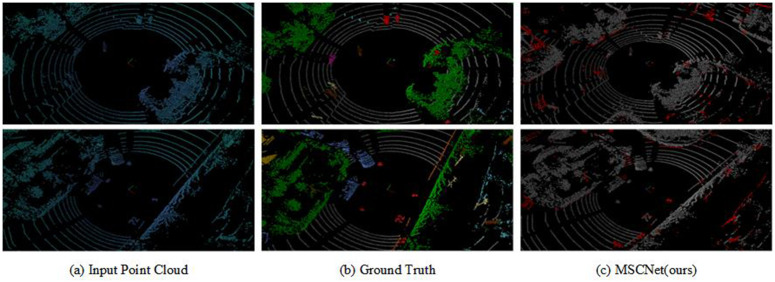
Qualitative analysis of the SemanticPOSS validation set. Where (a) and (b) are the input data and the corresponding segmented real data for the LiDAR scan frame, and (c) is the segmentation error map of our method in that scan frame. (Red colour indicates incorrect predictions).

To better qualitatively assess the differences between the predicted and labelled data, segmentation error maps were calculated, and the results are presented in Figs 5 and 6, respectively. Fig 5 shows the SemanticKITTI validation set (08 sequences), where Fig 5. (c) is an error map of the annotated data versus the MSCNet prediction results, which shows that our method produces high quality results in most categories (e.g., cars) and challenging categories (e.g., people, traffic signs), while the misclassified points (red points) include objects boundaries between objects and similar categories (e.g., buildings and fences).

Fig 6 shows the results of the validation set for SemanticPOSS, which contains more small moving objects (e.g., pedestrians, riders) and is more complex than the urban environment. Fig 6(c) shows the error plot calculated from the annotated data and the MSCNet predictions, which shows that our method produces high quality results in most categories (e.g., cars, plants) and challenging categories (e.g., people, riders), while the misclassified points (red points) are mainly objects that are far away from LiDAR.

### Ablation study

This section validates the hyperparameters of the loss function and the impact of each module in the network on the MSCNet using the 08 sequences in the SemanticKITTI dataset.

**Effect of hyper-parameter** λ. Table 4 illustrates the impact of the loss function hyperparameter λ on the segmentation performance (mIoU). It can be observed that as the value of λ increases, the model performance exhibits a clear trend of first increasing and then decreasing. When λ=0, meaning the intermediate supervision term is not activated and the model relies solely on the main loss function, the mIoU is 65.2%. After introducing intermediate supervision and setting λ=0.01, the mIoU significantly improves to 66.4%, indicating that the auxiliary loss begins to take effect. Further increasing λ to 0.05 and 0.1 leads to continued improvement in model performance, achieving mIoU values of 66.9% and 67.3%, respectively. The optimal result is obtained at λ=0.1, with a 2.1% improvement over the baseline, fully demonstrating the effectiveness of the intermediate supervision mechanism. However, when λ exceeds 0.1, the model performance begins to decline: at λ=0.5, the mIoU drops to 67.0%, and at λ=1.0, it further decreases to 66.6%. This phenomenon indicates that although intermediate supervision has a positive effect on model optimisation, its weight needs to be precisely controlled. If the weight is too small, the supervision effect cannot be fully utilised, while if it is too large, it disrupts the balance among the components of the loss function, thereby interfering with training. It is worth noting that within the relatively wide range of λ=0.05 to 0.5, the model maintains high performance, with a deviation of no more than 0.4% from the optimal result. Based on the above analysis, we set λ=0.1 as the optimal hyperparameter. Additionally, satisfactory results can be obtained within the range of λ=0.05 to 0.5.

**Table 4:** Effect of hyperparameter λ on the loss function.

λ	0	0.01	0.05	0.1	0.5	1.0
mIoU	65.2	66.4	66.9	67.3	67.0	66.6

**Impact of network modules.** In Table 5, we perform ablation studies to deeply analyze how the four modules (SCMF, DCR, PPM, FFM) jointly affect model accuracy, parameter count, and computational efficiency (Scan/sec). The first row shows the full model, which achieves an mIoU of 67.3%, an inference speed of 30 Scan/sec, and employs 1.6 M parameters. In the second row, we replace SCMFBlock with two 3×3 CBR blocks to remove SCMF’s contribution. After this replacement, inference speed increases to 32 Scan/sec, while mIoU drops to 65.8% (a loss of 1.5%). This indicates that although SCMFBlock introduces some computational overhead, it offers a substantial gain in representational power across channels. In the third row, we substitute the DCRBlock in the middle layers with a MobileBlock of similar parameter size. This replacement raises inference speed to 35 Scan/sec, but mIoU falls to 64.3%, a drop of 3.0%. This demonstrates that DCRBlock, despite its computational cost, plays a critical role in maintaining intermediate feature expressiveness and segmentation accuracy. In the fourth row, we replace PPM with a 3×3 CBR block with a comparable parameter count. As a result, inference speed slightly increases to 31 Scan/sec, but mIoU decreases to 64.2%, a drop of 3.1%. This suggests that the ability of PPM to capture global contextual information is vital for improving semantic segmentation accuracy, and its computational overhead is moderate, making it an efficient design choice. In the fifth row, we remove the feature concatenation and fusion operations within FFM, and only upsample and predict from the final output feature. This modification increases inference speed to 33 Scan/sec and slightly reduces the parameter count to 1.5 M, but the mIoU falls to 62.7%, a drop of 4.6%. This result indicates that the cross-layer feature fusion mechanism within FFM is crucial to the final segmentation accuracy, and its computational cost is justified by the performance gain. In summary, SCMF, DCR, PPM, and FFM each contribute to enhancing semantic segmentation accuracy to varying degrees. Although each module introduces some computational cost, together they strike an effective balance between accuracy and efficiency.

**Table 5:** Impact of each network module.

NO.	SCMF	DCR	PPM	FFM	Scan/sec	Param(M)	mIoU
1	✔	✔	✔	✔	30	1.6	67.3
2	✘	✔	✔	✔	32	1.6	65.8
3	✔	✘	✔	✔	35	1.58	64.3
4	✔	✔	✘	✔	31	1.6	64.2
5	✔	✔	✔	✘	33	1.5	62.7

### Effect of range on view resolution

As shown in Table 6, increasing the horizontal width W of the range view resolution leads to significant improvements in the model’s mIoU performance. However, this enhancement comes at the cost of reduced inference speed (scan/sec). Specifically, at lower resolutions (e.g.,64×512), the model operates at high speed but exhibits lower performance. In contrast, at mid-to-high resolutions (e.g., 64×2048), the model achieves an optimal balance between speed and accuracy, with the mIoU reaching its peak at 67.3. Further increases in resolution (e.g., 64×2560) result in diminishing returns, with performance gains plateauing or even slightly declining. This trend indicates that while higher resolutions enhance data completeness and segmentation accuracy, they also impose greater computational demands. To balance operational efficiency and segmentation precision, we have selected a resolution of 64×2048 for the range view in the SemanticKITTI dataset.

**Table 6:** Effect of range on view resolution.

Methods	Resolutions	Scan/sec	mIoU
MSCNet	64×512	85.2	60.5
	64 × 1024	68.7	63.3
	64 × 1536	47.5	64.5
	64 × 2048	30	67.3
	64 × 2560	15.5	67.2

### Multi-scale effects of the DCR block

To assess the contributions of each sub-module within the dilated convolution residual module (DCR) (whose architecture is illustrated in Fig 3) to the model’s final performance, we conducted a series of ablation experiments. Table 7 reports the mIoU scores under various combinations of sub-modules. When all sub-modules are kept, the model attains its highest mIoU of 67.3%, indicating that the full architecture can effectively fuse detail and context information across multiple scales. Removing the 1×1 CBR (the channel-mapping fusion branch) reduces the mIoU to 66.9% (a drop of only 0.4 points), which suggests that this branch, while beneficial for feature extraction, is not critical. Further removing the 3×3 CBR causes mIoU to fall to 66.5%, reflecting the important role of standard convolution in capturing local structural features. Eliminating the MaxPool branch (i.e., discarding the pooling scale transform) further decreases mIoU to 66.3%, indicating that feature aggregation play a positive role in improving the model’s scale generalization. Moreover, when only the MaxPool branch is retained, performance drops sharply to 62.5%; keeping only the 1×1 convolution yields an mIoU of 62.0%; retaining only the 3×3 convolution achieves 63.5%; and using only the dilated 3×3 convolution yields 64.5%. These results clearly demonstrate that relying on a single scale or branch severely limits the capacity for feature extraction. In summary, DCRBlock uses a multi-branch parallel structure to achieve complementary features, effectively mitigating information loss and thereby enhancing the generalization of the segmentation model across different scales and improving recognition accuracy.

**Table 7:** Multi-scale effects of the DCR block.

Module	1×1 CBR	3×3 CBR	3×3 DCBR	MaxPool	mIoU
DCR	✔	✔	✔	✔	67.3
	✘	✔	✔	✔	66.9
	✘	✘	✔	✔	66.5
	✘	✔	✔	✘	66.3
	✘	✘	✘	✔	62.5
	✔	✘	✘	✘	62.0
	✘	✔	✘	✘	63.5
	✘	✘	✔	✘	64.5

## Conclusion

In this paper, we propose an efficient and highly accurate network model, MSCNet, to accomplish real-time semantic segmentation of LiDAR data. The network can meet real-time requirements on GPUs. Due to the combined effect of SCMFBlock, DCRBlock, PPM, and FFM modules, MSCNet has better segmentation accuracy on small targets such as pedestrians and riders. And the best performance is achieved on SemanticPOSS and Pandaset datasets. The SCMFBlock performs multiscale feature extraction for each modal information individually, completing multimodal feature correction. The DCRBlock is located in the backbone network and uses dilation convolution to expand the perceptual field and reduce the network parameters. The PPM completes fast global feature acquisition. The FFM is located at the end of the network and completes the fusion of high-level and low-level features. In addition, an intermediate layer of supervision using coaching allows the network to converge quickly. We evaluated the method on SemanticKITTI and showed that MSCNet better balances parametric number, speed, and accuracy. However, the projection-based approach has some limitations. For example, projection leads to the loss of information. We will investigate such issues in future work.
